# Can Text Messages Increase Empathy and Prosocial Behavior? The Development and Initial Validation of Text to Connect

**DOI:** 10.1371/journal.pone.0137585

**Published:** 2015-09-10

**Authors:** Sara Konrath, Emily Falk, Andrea Fuhrel-Forbis, Mary Liu, James Swain, Richard Tolman, Rebecca Cunningham, Maureen Walton

**Affiliations:** 1 Lilly Family School of Philanthropy, Indiana University, Indianapolis, IN, United States of America; 2 Institute for Social Research, University of Michigan, Ann Arbor, MI, United States of America; 3 Department of Psychiatry, University of Rochester Medical Center, Rochester, NY, United States of America; 4 Annenberg School of Communication, University of Pennsylvania, Philadelphia, PA, United States of America; 5 Department of Psychiatry, Psychology, and the Center for Human Growth and Development, University of Michigan, Ann Arbor, MI, United States of America; 6 School of Social Work, University of Michigan, Ann Arbor, MI, United States of America; 7 Department of Emergency Medicine, University of Michigan, Ann Arbor, MI, United States of America; 8 Injury Center, University of Michigan, Ann Arbor, MI, United States of America; University of Vienna, AUSTRIA

## Abstract

To what extent can simple mental exercises cause shifts in empathic habits? Can we use mobile technology to make people more empathic? It may depend on how empathy is measured. Scholars have identified a number of different facets and correlates of empathy. This study is among the first to take a comprehensive, multidimensional approach to empathy to determine how empathy training could affect these different facets and correlates. In doing so, we can learn more about empathy and its multifaceted nature. Participants (N = 90) were randomly assigned to receive either an empathy-building text message program (*Text to Connect*) or one of two control conditions (active versus passive). Respondents completed measures of dispositional empathy (i.e. self-perceptions of being an empathic person), affective empathy (i.e. motivations to help, immediate feelings of empathic concern), and prosocial behavior (i.e. self-reports and observer-reports) at baseline, and then again after the 14 day intervention period. We found that empathy-building messages increased affective indicators of empathy and prosocial behaviors, but actually *decreased* self-perceptions of empathy, relative to control messages. Although the brief text messaging intervention did not consistently impact empathy-related personality traits, it holds promise for the use of mobile technology for changing empathic motivations and behaviors.

## Introduction

*“Every smallest stroke of virtue or of vice leaves its never so little scar*.*”*

~William James, 1914, Habit

Enduring and compassionate social bonds are fundamental to human health and well-being [1, 2]. Yet, in recent years, there have been documented declines in face-to-face social interactions, and in young people’s self-perceived ability to care and connect with others [3–8]. Some scholars have suggested that new media and technologies at least partially account for such changes [9, 10], but the use of new media can also be associated with building social connections and social capital [11, 12].

In this paper, we examine whether it is possible to causally increase empathic feelings, motivations, behaviors, and traits using one such new technology (i.e., text messages on mobile phones) in young adults. Can we use mobile technology to nurture empathic habits? If so, this would have the potential to reach a lot of people with very little resources. At first glance, this seems like a simple applied problem: give people daily reminders to be empathic (versus a control message) and then later test whether this works. However, this study is among the first to take a comprehensive, multidimensional approach to empathy in order to determine how empathy training could affect a number of different facets and correlates of empathy, using both established and novel measures and conceptualizations. In doing so, we can learn more about empathy and its multifaceted nature.

Empathy is very difficult to define and measure; the scholarly literature on it is full of different conceptualizations and operationalizations of it. Scholars have used the term loosely to apply to personality traits [13, 14], emotional responses [15], cognitive states or abilities [16–19], and hypothetical responses to situations [20, 21]. Thus, a general definition that encompasses this broad literature is that empathy involves *a focus on and concern for others’ perspectives and feelings* [22]. The scholarly literature is much more consistent in identifying the correlates and consequences of empathy. The empathy-altruism hypothesis has been clearly supported by a number of researchers: people who score high in trait empathy [23], or whose empathic feelings have been temporarily activated, are more likely to help others [15].

In trying to better understand this complex literature, we see three important facets to consider. First, one of the most critical aspects of empathy is the affective part: both the tendency to be emotionally moved by other people’s situations (emotional) and the desire to help others (motivational). These more affective parts seem to be critical to inspiring a number of desirable behavioral outcomes, such as an increase in prosocial behaviors [15, 23–25] and a decrease in aggression [26, 27].

Second, it is also important to study behavioral implications of these empathic emotions and motivations. Ideally, an empathy training program will have the broader impact of increasing prosocial behavior, in addition to increasing various facets of empathy, whether immediate situational responses to others’ in need or more chronic empathic tendencies. Yet prosocial behavior does not tell us much in itself. For example, people might volunteer for nonprofit organizations because they want to feel good, rather than because they care about helping others or feel for those in need [28]. Thus, behavioral outcomes should not be the only signals of prosociality that researchers assess when studying the efficacy of an empathy training program. At the same time, it would be equally inappropriate to simply assess people’s empathic motivations and emotions, without examining whether behavioral change also occurred. People can have good intentions and concerned feelings for others without acting on their impulses. The fact that empathy motivates prosocial behavior and inhibits aggressive behavior is one of the reasons that it is so highly valued in society [15, 23–27]. To further complicate the matter, there are different kinds of prosocial behaviors. Some are more closely tied to empathic activation (e.g. helping others who are in distress) while others are more peripheral forms of prosocial behavior (e.g. cooperation with others) that nevertheless have been found to be correlated with empathy [29]. It is important to test the potential scope of empathy training programs to see how far they generalize outside of a specific target in distress.

Finally, the most common way that empathy is assessed within the literature is via self-reported trait measures [14, 30, 31]. One commonly used multidimensional measure of empathy, the *Interpersonal Reactivity Index*, includes affective other-focused (Empathic Concern), cognitive other-focused (Perspective Taking), and affective self-focused (Personal Distress) components [14]. There is evidence for the reliability and validity of these standardized instruments, but at the same time, traits are essentially self-perceptions, which are subject to a number of distortions. For example, people sometimes have a lack of self-awareness about their personal traits, or are motivated to respond in ways that make them look good [32–34]. Self-perceptions may also change, depending on the context. For example, after seeing an image of a highly attractive person, people judge themselves as less attractive [35]. More relevant to the current paper, one recent study found that exposure to a highly prosocial exemplar (i.e. Superman) led to *lower* helping intentions [36]. Participants could only contrast themselves to such a high standard. Traits are important indicators of empathy, to be sure, but in order to be convinced that a person is empathic, it would also be important to demonstrate that they feel more compassionate emotions for others, want to help others, and actually do so when there is a need and they are given the opportunity to help.

Is empathy malleable? There is evidence that empathic traits are relatively stable, but also, that they can be responsive to people’s general environments and immediate situations. Empathic traits are at least partially heritable, with approximately 50% of the variation in such traits explained by genetic factors [37–40]. People who exhibit more empathic tendencies at one time, also tend to show more of these tendencies between several months and several years later [41–48]. Indeed, one study found that empathic traits were positively correlated across a 17 year period, from preschool to young adulthood [49].

Yet empathic traits are also at least partially changeable. Since genetic factors only explain half of the variation in such traits, this implies that the other half is explained by environmental factors. Moreover, although there is cross-temporal and cross-situational consistency in empathic tendencies, the effect sizes are small enough to leave room for individual change in such tendencies.

To this end, empathic predispositions are at least partially driven by motivation and practice. For example, people who are more motivated, whether intrinsically or extrinsically, can increase their empathic feelings and accuracy [50, 51]. This demonstrates that empathy is at least partially under conscious cognitive control. That is, people can choose to be more or less empathic, at least in certain circumstances. In addition, parenting practices that encourage children to take others’ perspectives and discourage aggression predict higher empathy and more prosocial behavior in children, suggesting an environmental role [52–55]. Moreover, our recent work has found that college students have been declining over time in dispositional empathy over the past 30 years [7]. Temporal trends such as these suggest that broad sociocultural factors can affect empathic tendencies.

Socio-emotional intervention programs have also been shown to affect empathy. For example, empathy can increase when people are taught to notice the ways in which they are similar to others [56, 57], when they are taught better emotion recognition abilities or interpersonal skills [58, 59], when they practice imagining or role-playing other peoples’ experiences [24, 60–62], when they observe others’ misfortunes [63, 64], and after observing others’ empathic or generous acts [59, 65, 66].

However, few studies have experimentally induced empathy in longitudinal, real world contexts to examine the causal role of regularly generating empathic states on subsequent empathic motivations, emotions, self-perceptions, and prosocial behaviors. Some theorists consider more automatic (effortless) versus more controlled (effortful) aspects of empathy [67]. We posit that if people repeatedly engage in practicing empathizing, this will ultimately lead to more automatic (or habitual) empathic responses. This is in line with research demonstrating that tasks that initially take much concentration can later become automatic, almost done without thinking [68, 69]. Indeed, higher empathy people exhibit spontaneous muscle mimicry of facial expressions when stimuli are presented below their conscious awareness [70]. This provides some support for the idea that empathy may become more automatic with expertise.

### Text messaging as interventions

Many prior empathy-induction studies rely on classroom or laboratory teaching, yet some studies have found that empathy can be taught using videos [59, 71]. This demonstrates that it may not be necessary for empathy-building programs to be face-to-face in order to be effective.

In contrast to prior approaches, text messaging is simple, low cost and ubiquitous, but underutilized in psychosocial domains [72]. Approximately 9/10 Americans own a mobile phone [73], and 6/10 of those are smartphones [74]. Mobile phones are often kept close to people, checked several times throughout the day, and have acquired learned associations with interpersonal intimacy [75, 76]. One study found that people had their phones in the room with them 90% of the time, and 50% of the time they were within arm’s reach [77].

Much research in public health has found that it is possible to use text messages to help people manage disease (e.g. diabetes) and make better health decisions such as smoking less and exercising more [78–87]. Text message interventions are inexpensive to implement, can be embedded within participants’ everyday lives, and can be widely and broadly disseminated. Yet, so far the research using text messaging as an intervention has predominantly been focused on trying to change health-behaviors, with virtually no mobile-based research trying to change psychological traits or social behaviors [72].

Can we use text messages to increase empathy? Some scholars have argued that new media, including mobile phones, may diminish empathy and social capacities [9, 10]. For example, one experiment found that simply having a cell phone in the room (versus a control object: a book) reduced participants’ empathy and feelings of closeness and trust during a social interaction [88]. Another study found that being reminded of or using their cell phone activated fewer prosocial thoughts and made people less likely to help others in need [89].

Although mobile phones may distract from face-to-face interactions under some circumstances, they also literally connect us to others, making us feel closer to loved ones when there is physical distance [75, 76, 90–93]. With the exception of the two experiments described above, most prior research on mobile phones and social connectedness has correlated natural usage patterns with social connectedness variables.

### Current study

The current study asks if it is possible to increase empathic motivations, traits, and behaviors through simple daily reminders. By extension, can we transform mobile phones into psychosocial intervention tools to increase empathic capacity? If so, what can we learn about the multifaceted nature of empathy? Young Americans (aged 18 to 24) send and receive over 125 text messages per day [94]. This makes text messaging a commonplace and likely powerful mode of intervention among an age group that has relatively lower trait empathy compared to other Americans [7, 8].

This paper describes the development and validation of a simple one-way text messaging program, *Text to Connect*, which is designed to both increase empathy-related outcomes among young adults, and to further understand the multifaceted nature of empathy. We used a theoretically driven approach to build participants’ empathic capacity, which interspersed training on emotional and cognitive aspects of empathy with reminders to act prosocially. Our rationale was that daily practice in feeling with others, imagining their perspectives, and doing small kinds acts may help to create empathic habits in young adults. We conducted a small (N = 90) randomized control trial to examine how this empathy-building program altered several different facets of empathy (i.e. affective, general beliefs, traits / self-perceptions, and behaviors).

## Method

### Part 1: Development and selection of text messages for Text to Connect

In order to develop Text to Connect, we drew on prior empathy intervention research, and also on items from empathy and narcissism scales, to write an initial pool of 39 higher empathy text messages and 38 lower empathy ones. The low empathy (active control) messages were designed to be low in empathy in one of two ways: some of them focused on increasing self-esteem (versus esteem for other people), and others encouraged more objectivity or psychological detachment (versus emotional engagement). Each message was 140 characters or less and was written in everyday language. See S1 Table for the complete list of messages.

Next, the 77 messages were presented to a group of 22 raters in a randomized order, and the raters answered questions for each message that they saw. The raters were all affiliated (undergraduate research assistants, graduate students, postdocs, and faculty) with the first author’s research lab. The raters were not the same people who originally developed the messages.

For the non-behavioral (cognitive, emotional) messages, raters were asked the following two questions:

1. How other-oriented (caring, empathetic) versus self-oriented (egotistical, individualistic) is this statement? (1 = *extremely self-oriented*, 3 = *neutral*, 5 = *extremely other-oriented*).
2. How logical (based on thinking, cool) versus emotional (based on feelings, warm) is the statement? (1 = *extremely logical*, 3 = *neutral*, 5 = *extremely emotional*).

For behavioral messages, raters were asked the following two questions:

1. How other-oriented (caring, empathetic) versus self-oriented (egotistical, individualistic) is this statement? (1 = *extremely self-oriented*, 3 = *neutral*, 5 = *extremely other-oriented*).
2. How much is this just in someone’s mind or thoughts versus their behavior? (1 = *definitely only in the mind*, 3 = *neutral*, 5 = *definitely action / behavior*).

Using these ratings, we selected 21 messages (8 cognitive, 8 emotional, 5 behavioral) in each condition (high empathy versus low empathy) that met the following criteria:

#### 1) High versus low empathy conditions

To be included in the high empathy condition, messages had to score above the midpoint (3) in Self/Other ratings. To be included in the low empathy (active control) condition, messages had to score below the midpoint in Self/Other ratings. Indeed, the 21 messages chosen to be in the high empathy condition were higher in Self/Other ratings (*M* = 4.46) than the 21 low empathy messages (*M* = 1.83), *F*(1,40) = 251.60, *p* < .001. (See Column F in Table 1 for mean ratings.)

**Table 1:** Characteristics of text messages used in experiment.

A. Condition	B. Type	C. Mean Number of Characters	D. Mean Grade Level	E. Mean Reading Ease	F. Mean Rater Self (1) to Other (5)	G. Mean Rater Logical (1) to Emotional (5)	H. Mean Rater Mind (1) to Behavior (5)
High Empathy	Emotional	106.00	4.41	80.21	4.57	4.17	—
High Empathy	Cognitive	136.13	5.20	74.24	4.40	2.55	—
High Empathy	Behavioral	88.40	4.68	80.70	4.40	—	4.45
	**Overall**	**110.18**	**4.76**	**78.38**	**4.46**	**3.36**	**4.45**
Low Empathy	Emotional	111.25	5.31	77.24	1.51	3.47	—
Low Empathy	Cognitive	131.13	6.58	69.79	2.36	1.84	—
Low Empathy	Behavioral	89.00	6.64	67.96	1.62	—	4.25
	**Overall**	**110.46**	**6.18**	**71.66**	**1.83**	**2.67**	**4.25**

#### 2) Emotional, cognitive, and behavioral items

Messages that were rated above the midpoint (3) for the Logical/Emotional question were categorized as emotional, and messages that were rated below the midpoint were categorized as cognitive. These categorizations were effective within both the high empathy and the low empathy conditions. Within the high empathy condition, the emotional messages were rated as significantly more emotional (*M* = 4.17) than the cognitive messages (*M* = 2.55), *F*(1,14) = 120.75, *p* < .001, and within the low empathy condition, this was also true (*M*_emotional_ = 3.47; *M*_cognitive_ = 1.84), *F*(1,14) = 33.29, *p* < .001. (See Column G in Table 1 for mean ratings.)

Messages that were rated above the midpoint (3) for the Mind/Behavior question were categorized as behavioral. Messages were rated as equally behavioral in the high empathy (*M* = 4.45) and the low empathy conditions (*M* = 4.25), *F*(1,8) = .15, *p* = .71. (See Column H in Table 1.) All three types of messages were presented to participants in each condition.

By design, the number of characters per text message was similar across the high empathy (*M* = 110.18) versus low empathy conditions (*M* = 110.46), *F*(1,36) = .002, *p* = .97. Yet across both conditions, the longest messages were the cognitive ones (*M* = 133.63), then the emotional ones (*M* = 108.63), and the behavioral ones were the shortest (*M* = 88.70), *F*(2,36) = 15.58, *p* < .001. Importantly, there was no interaction between condition and message type, *F*(2,36) = .25, *p* = .78.

Although we tried to match the messages across conditions in terms of reading difficulty, the text messages in the high empathy condition (*M* = 4.76) were at a marginally lower reading grade-level compared to those in the low empathy control condition (*M* = 6.18), as demonstrated by the Flesch-Kincaid Grade, *F*(1,36) = 3.07, *p* = .089, which represents the US grade reading level. However, another readability metric (Flesch-Kincaid Reading Ease) found no significant difference across the two conditions, *F*(1,36) = 2.53, *p* = .12 (See Table 1, Columns D and E). Moreover, the fact that the control condition grade level was still quite low (i.e. 6th grade), makes these messages very accessible, especially to the current sample.

### Part 2: Examining the effect of Text to Connect

#### Design

**Fig 1:**
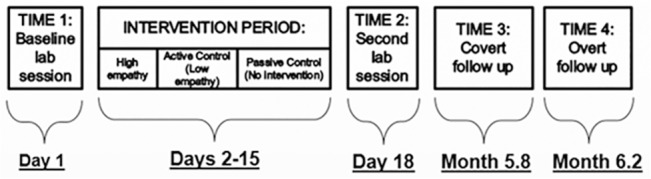
Overview of study design.

This study used a pre-post longitudinal experimental design, with three between-subjects conditions. See Fig 1 for a schematic diagram of the study.

#### Ethics statement

This study was approved by the Institutional Review Board at the University of Michigan. Participants provided written informed consent at the beginning of the study. Since participants did not give their consent to post their data online, we cannot do so. However, we will share a de-identified data set with other academic researchers who have obtained IRB approval from their institution to analyze these data. These researchers will be required to sign a data confidentiality and protection agreement. Please email the first author for data requests.

#### Participants

Participants in the baseline lab session (Time 1) were 90 college students (60% female) with a mean age of 20.98 (SD = 4.29), who participated in exchange for a monetary incentive. Participants were paid up to $100 for their time: $12 for a baseline lab session, 30 cents per response to each daily mood measure, plus $2.70 if they responded to all six of them, and $25 for a follow-up lab session.

The ethnic composition of the sample was 52% Caucasian, 33% Asian, 11% African-American, and 4% Other or Unidentified. Seven participants did not return for the follow-up lab session (Time 2). This left a final sample size of 83/90 (92.2%; 60% female; Mean age = 20.93, SD = 4.33), with 51% Caucasian, 34% Asian, 12% African-American, and 3% Other or Unidentified. Of those, 4 participants declined to be further followed up and thus we did not contact them for later parts of the study. All data were collected between November 2012 and August 2013.

#### Procedure

Participants were told that they were participating in a social skills training study. At baseline (Time 1) participants completed a packet of questionnaires measuring empathy-related traits, behaviors, and motives, embedded within a larger set of measures assessing health and wellbeing to be reported elsewhere.

Participants were randomly assigned to one of three intervention conditions: High Empathy (Text to Connect; N = 37), Low Empathy Control (N = 21), and No Intervention Control (Responses only; N = 25). Because of limited resources, we deliberatively weighted the randomization so that there would be more participants in the high empathy condition compared to the other two conditions (Combined N = 46). This was so we could examine overall effects of empathy training compared to any control condition.

The intervention phase of the study began the day after the baseline lab session. All participants also received text messages 6 times a day for 14 days asking them to rate their mood, feelings of connectedness, and social interactions. Responses to these questions were incentivized and on average, participants responded to 76.6 of the 84 messages (91.2%) across the 14 day period. (This number was 78.2 for those who completed the follow up lab session.) These messages always came 5 minutes after intervention texts, if applicable. Data from this ecological momentary assessment part of the study will be reported elsewhere.

#### Post-intervention assessment (Time 2)

Participants returned to the lab an average of 19 days (SD = 5 days) after the baseline lab session and completed questionnaires and procedures nearly identical to the baseline (see measures section below). A logistic regression found that there was no effect of condition on the tendency to dropout (1 = yes, 0 = no) at the post-intervention session, *p* = .99.

#### Covert follow-up (Time 3)

An average of 5.8 months (range: 3.8 to 8.5 months) after the second lab session, we conducted a covert follow-up assessment that involved sending participants a hostile text message (“*stop txting me u jerk*!”) and then recording their verbatim responses. Forty-three out of the 79 contacted participants (54.4%) responded to this message; 50% of these responses were received within the first 5 minutes (Mean = 76 minutes after receiving text). A logistic regression found that there was no effect of condition on the tendency to respond to the text message, *p* = .46.

#### Overt follow-up (Time 4)

We conducted an overt follow-up assessment via an online survey two weeks after the covert follow-up text message. This occurred an average of 6.2 months (SD = 1.5 months) after the second lab session. Sixty out of the 79 contacted participants (75.9%) completed this survey. A logistic regression found that there was no effect of condition on the tendency to complete the overt follow-up survey, *p* = .93.

### Immediate Post-Intervention (Time 2) Measures

All empathy measures and outcomes fit into the categories of i) *Affective* (i.e. motivations and emotions), ii) *General beliefs*, iii) *Traits / self-perceptions* (i.e. chronic and stable dispositions), and iv) *Behaviors* (self-reported and observed). In the Results section and S2 Table, we thus categorize them as such to help organize their interpretation.

#### Motives for volunteering

At both the baseline and post-intervention sessions, participants reported whether they currently volunteered for non-profit organizations, and listed the reasons why. We coded the reasons into three categories. One category was other-oriented: to help others (1 = *yes*, 0 = *no*), and two were more self-oriented: because it feels good (1 = *yes*, 0 = *no*), and to help participants’ career or to learn something new (1 = *yes*, 0 = *no*).

#### Aggressive beliefs

We measured aggressive beliefs using the Normative Beliefs About Aggression Scale [95], which assesses the extent to which people believe that is okay to behave aggressively under various circumstances, including direct provocations. An example item is: “*Suppose a young man hits another young man*, *John*. *Do you think it’s wrong for John to hit him back*?” (1 = *It’s really wrong*, 2 = *It’s sort of wrong*, 3 = *It’s sort of OK*, 4 = *It’s perfectly OK*). The 20 items were averaged, with higher numbers indicating more aggressive beliefs.

#### Moral principle of care

The eight item Principle of Care scale [96, 97] measured participants’ beliefs about the moral importance of caring for others. A sample item is: “*People should be willing to help others who are less fortunate*” (1 = *strongly disagree*, 3 = *neutral*, 5 = *strongly agree*). Items were averaged for analysis, with a higher score indicating more caring attitudes.

#### Dispositional empathy

We assessed four dimensions of trait empathy with the 28 item Interpersonal Reactivity Index [14]. Participants rated each item from 1 (*does not describe me well*) to 5 (*describes me very well*). Perspective Taking, or cognitive empathy, involves imagining others’ perspectives (e.g. “*I sometimes try to understand my friends better by imagining how things look from their perspective*”), and Empathic Concern, or emotional empathy, involves feeling compassion for others (e.g. “*I often have tender*, *concerned feelings for people less fortunate than me*”). Fantasy involves an imaginative identification with fictional characters (e.g. “*I really get involved with the feelings of the characters in a novel*”), and Personal Distress involves more self-oriented emotional responses to others’ painful experiences (e.g. “*When I see someone who badly needs help in an emergency*, *I go to pieces*”). Items were averaged, with a high score indicating greater dispositional empathy.

#### Giving and receiving social support

Participants answered eight questions about giving and receiving support. Two of the questions were general: “*During the past seven days*, *was anyone available to help you [were you available to help others] with advice*, *encouragement*, *moral or emotional support*?” The other six questions used a similar format to ask whether participants had given or received emotional support from 1) friends, neighbors, or co-workers, 2) brothers or sisters, and 3) parents. We calculated a ratio by dividing the sum of the giving support questions by the sum of the receiving support questions. Thus, numbers above one indicate giving support more than receiving support, and numbers below one indicate receiving support more than giving support.

#### Empathy in imagined scenarios

Participants read three short scenarios describing a friend in distress (available upon request to the first author), and then were asked to write the exact words that they would say in response to their friend. One scenario described a friend who had studied very hard for a test but got a low grade. The friend said that he felt overwhelmed about an upcoming paper. The second scenario described a friend who was arrested after accidently putting toothpaste into her pocket when her arms were full with other items. The last scenario described a friend who thought her boyfriend might be cheating on her because she discovered a suspicious text message. All three of the scenarios included the friend directly sharing negative emotions with the participant. Participants’ written responses were coded by blind coders for the presence of four themes. Two assessed *emotional empathy*: e*motional resonance*, when participants echoed their imagined friend’s emotional experiences (1 = *yes*, 0 = *no*); and *emotion acknowledgment*, when participants acknowledged their friend’s emotional experiences (1 = *yes*, 0 = *no*). These were summed across the three scenarios into an emotional empathy score. Two assessed *practical empathy*: *offering to listen* to their imagined friend if needed, and *offering to help* in some practical way. These were summed into a practical empathy score. Responses from the baseline and post-intervention lab sessions were coded by one main coder and one reliability coder. Inter-rater reliability for practical empathy (α_baseline_ = .84; α_post-intervention_ = .92) was higher than for emotional empathy (α_baseline_ = .54; α_post-intervention_ = .51), perhaps because the practical acts were more concrete than the emotional ones. Because of the lower internal reliability of the emotional empathy subfactor, these results should be interpreted with caution.

#### Situational empathic emotions and behavioral intentions to help

After completing the self-report questionnaires, at the end of both lab sessions, participants were exposed to someone in distress, and then they were given an opportunity to help the individual. Participants’ responses to these helping tasks can be considered behavioral intentions to help, since we did not actually follow up with participants who offered to help.

In the baseline lab session, we used the classic Katie Banks task, in which participants listened to a radio story about a student whose parents just died in a car accident [98, 99]. The radio host mentions that the radio station will be assisting with organizing volunteers for her. After listening to the program, participants were given a letter supposedly handwritten by Katie, and a form from the local radio station which asked them if they were willing to volunteer, and if so, for how many hours. Participants sealed their responses in an envelope addressed to the local NPR session, and thus, research assistants were unaware of participants’ responses. We told participants that their letters would be mailed to the radio station.

In the post-intervention lab session, participants watched a short video of Karen Klein, a school bus monitor, being bullied by adolescent boys. This was a real situation that involved the boys bullying Karen, videotaping it, and posting the video to YouTube. The bullying is quite severe and involves name calling, swearing, and threats of sexual assault. The original video was 10 minutes long, and we reduced it to just over 2 minutes for the purpose of this study. In the video, Karen is clearly in distress, and starts crying at one point. We told participants that we were partnering with a local anti-bullying organization, and they were given a brochure from the organization in addition to a volunteer sign up form that asked if they were willing to volunteer, and if so, for how many hours. As before, participants sealed their responses in an envelope, and thus, research assistants were unaware of participants’ responses. We told participants that their letters would be mailed to the anti-bullying organization.

Note that research assistants were blind to experimental condition and thus did not know whether participants were in the high empathy or control conditions.

#### Immediate emotional responses to targets in distress

During both lab sessions, we assessed *personal distress* emotions by asking participants to what extent they felt distress for themselves (4 items: e.g. “*directly distressed*, *as if I personally experienced something bad*”; 1 = *very slightly*, 5 = *extremely*; α_baseline_ = .95; α_post-intervention_ = .95), and *empathic* emotions by asking participants to what extent they felt distress for the individual in distress (4 items: “*distressed for the person being interviewed*”; α_baseline_ = .95; α_post-intervention_ = .86).

#### Observer-reported empathy

Research assistants who were blind to study condition rated participants’ empathy immediately after interacting with them. Specifically, they were asked: *“To what extent do you think this statement describes this person*: *‘He or she is an empathetic person*.*’”* (1 = *not very true of him or her*; 7 = *very true of him or her*). No further instructions were given and there was no inter-rater reliability since research assistants administered the study alone. Thus, results should be considered exploratory, and future research should include a more rigorous protocol for rating participants’ empathy.

### Covert follow-up (Time 3) Measures

As mentioned, research assistants were blind to participants’ condition during the lab sessions. However, after the 14 day intervention, it was possible that participants who received text messages might have had ideas of what we were trying to study. For example, participants in the empathy training condition could have responded more prosocially because of demand characteristics. Thus, we ran an additional test of our hypothesis in which participants themselves were also blind to condition. We devised a behavioral test of prosociality that did not appear to be related to the researchers or the study. Since we had already assessed specific responses to distressed others during the post-intervention lab session (Time 2), we moved to a more peripheral measure for the covert follow-up (Time 3). The rationale was that since research finds that empathy is associated with less aggressiveness and more cooperativeness [27, 29], people who are trained to be more aware of others’ needs may respond in a less aggressive / more prosocial way to a rude text message from a stranger.

Approximately 5.8 months after the second lab session, we sent participants a hostile text message from a cell phone number that they did not know and could not be identified by an internet search. The text message said: “*stop txting me u jerk*!” We recorded participants’ verbatim responses and two independent coders coded these responses for prosociality (interrater reliability: α = .95). More aggressive responses were coded as 1 (e.g. “*Never*!” and “*I feel like it is completely within my right to continue*”), neutral responses were coded as 2 (e.g. “*Who is this*?*”*), and more prosocial responses were coded as 3 (e.g. “*I’m sorry you’re having a bad day*, *but I think you have the wrong number*”). In other words, higher numbers indicated more prosocial responses, and lower numbers indicated less prosocial responses.

### Overt follow-up (Time 4) Measures

The overt follow-up took place approximately 2 weeks after the covert follow-up phase.

#### Motives for volunteering

In the overt follow-up, motives for volunteering were measured with a standardized scale rather than open-ended responses. Two items from each of the six Volunteer Functions Inventory subscales [28, 100] were included in the survey. As in prior research [101], an *other-oriented motives* scale (α = .66) was created by combining the Altruistic Values (e.g. “*I feel compassion toward people in need*”) and Social Connection (e.g. “*Others with whom I am close place a high value on community service*”) subscales. A *self-oriented* motives scale (α = .71) was created by combining the Career (e.g. “*I can make new contacts that might help my business or career*”), Self-Protection (e.g. “*Volunteering helps me work through my own personal problems*”), Self-Enhancement (e.g. “*Volunteering makes me feel better about myself*”), and Learning/Understanding (e.g. “*Volunteering lets me learn through direct ‘hands on’ experience*”) subscales. For all items 1 = *Is definitely not a reason that I volunteer*, and 7 = *Is definitely a reason that I volunteer*.

#### Feelings of social connectedness

Participants were asked to report how connected to others they felt right now (1 = *not at all*, 2 = *a little bit*, 3 = *moderately*, 4 = *fairly*, 5 = *extremely*). We explained that being connected to others means feeling emotionally close to people, whether or not participants were actually with them right then.

#### Dispositional empathy

We again used the Interpersonal Reactivity Index [102] to assess empathic traits, with one minor modification. It is possible that receiving the empathy messages might have created an exceptionally high standard for empathic behavior and altered participants’ reference groups when they answered the IRI questions (see Results and Discussion for more information). In other words, participants may be drawing on a wide range of exemplars, including an abstract ideal of a highly empathic person, when rating themselves on empathy. In order to counteract this possibility, we began each item in the IRI with “*Compared to other people my age*,” and then continued with the verbatim item. This served to limit the potential range of comparison targets to a familiar group of people, rather than an abstract ideal of high empathy.

#### Number of in-person social interactions

Participants were asked to report the number of people they talked to in the past 2 hours. We asked them about the most recent 2 hour period in order to limit potential recall bias. We specified that this should include anyone they knew, regardless of closeness, but should not include people they do not know (e.g. service employees, strangers). Although this measure does not assess empathy or prosocial behavior, we included it because we wondered whether empathy training would have the spillover effect of increasing the motivation to interact with people. Since our program was designed to increase empathic responding in the context of current social relationships, we thought it was possible that people in the empathy training program would want to spend more time with others after practicing some of the empathic skills.

#### Giving and receiving social support

We used the same measures as in the baseline and post-intervention survey to create the giving:receiving support ratio.

#### Social dilemma game

We aimed to examine the potential scope of *Text to Connect* by not only directly assessing different operationalizations of empathy, but also by examining broader theoretically relevant outcomes that are known to be associated with empathy. Previous research has shown that people who are randomly assigned to empathize with someone (versus remain objective) are more likely to cooperate in social dilemma games [29]. Thus, we examined whether Text to Connect influenced people’s behavioral responses in a social dilemma game.

Participants were told that they could earn additional money (up to $10) as a bonus from playing an economics game. We told them that economics games are those in which individuals make decisions with another actual person, not necessarily at the same time or from the same location. We said these games are often played online or at different times and locations, and all the game needs is two different people who are making the same decision. We also told them that in this game they would be randomly paired with one other participant from this study. Participants would be matched with the participant who responded to the survey either right before or right after them (selected by a coin flip). We asked participants to make a choice after seeing a table that presented the four options in the game. If they and their partner both cooperated, both would get $6 bonuses. If one partner defected and the other cooperated, the defector would get $10 and the cooperator would get $0. And if both participants defected, they would both receive $0.

## Results

### Data analysis strategy

Because of our limited sample size and our interest in the effect of empathy training (N = 37) relative to other conditions, we oversampled participants to be in the high empathy condition, and then collapsed across the two control conditions (Low Empathy: N = 24; No Message Control: N = 29). Randomization was weighted so that approximately 40% of participants would be in the high empathy group, and 30% in each of the other groups. Because of the small sample size in this pilot study, we also report marginally significant results (p < .10), and are careful to interpret these results with caution. We include Cohen’s D effect sizes in S2 Table to help readers more accurately interpret the results.

Because empathy and self-esteem are influenced by gender [8, 103, 104], it is important to examine whether men and women differentially respond to the training. Note that the specific number of participants included in each analysis varied because some participants did not complete all measures. Baseline measures were entered as covariates whenever applicable. We conducted a 2 (Condition: Empathy versus Combined Control) × 2 (Gender: Men versus Women) between-subjects analysis of covariance (ANCOVA) on each post-intervention and follow-up dependent variable, controlling for baseline scores whenever applicable. When baseline measures were not available as covariates, a Condition × Gender ANOVA was conducted instead. Since there was no effect of condition on dropout rates, dropout cases were treated as missing for analyses. Logistic regressions were conducted in the case of categorical dependent variables, with the same predictors.

### Immediate Post-Intervention (Time 2) Measures: Affective (motives and emotions)

#### Motives for volunteering

Most (86%) participants were involved in extracurricular volunteering activities. We examined the effect of Condition and Gender on the reasons that participants volunteered (or would volunteer), controlling for baseline levels of their motives. A main effect of condition found that participants in the high empathy condition (M = 88%, SE = 7%) were more likely to report that they volunteered (or would volunteer) because of other-oriented reasons (i.e., they wanted to help others) compared to those in the control conditions (M = 69%, SE = 6%), *β* = 2.20, *p* = .03, O.R. = 9.01, C.I.[1.20–67.71]. There was no main effect of Gender, *β* = 1.26, *p* = .17, nor was there an interaction, *β* = 18.40, *p* = .99.

Participants in the high empathy condition (*M* = 28%, *SE* = 8%) were *less* likely to report that they volunteered (or would volunteer) because of self-oriented reasons (i.e., it feels good) compared to those in the control conditions (*M* = 49%, *SE* = 6%), *β* = -1.61, *p* = .03, O.R. = 0.20, C.I.[0.05–0.82]. There was no main effect of Gender, *β* = -0.30, *p* = .66, nor was there an interaction, *β* = 0.95, *p* = .49. Although women (*M* = 31%, *SE* = 5%) were more likely than men (*M* = 7%, *SE* = 7%) to say that they volunteered to further their career, *β* = -2.31, *p* = .02, O.R. = 0.10, C.I.[0.02–0.65], there was no effect of Condition, *β* = -0.74, *p* = .36, or interaction, *β* = -18.76, *p* = .99.

#### Emotional responses to targets in distress

Two separate 2 (Condition) × 2 (Gender) ANCOVAs were conducted on post-intervention *personal distress emotions* and *empathic emotions in response to targets in distress*, controlling for baseline levels.

Although the baseline helping task had a different target in distress than the Time 2 (immediate post-intervention) target (i.e. Katie Banks at baseline versus Karen Klein at Time 2), those who felt more compassionate emotions in response to Katie also felt more compassionate emotions in response to Karen (*r* = .49, *p* < .001). A similar pattern was found for personal distress emotions (*r* = .34, *p* = .002). This suggests that there are individual differences in the tendency to emotionally respond to such stimuli. Thus, we controlled for baseline scores in this analysis.

When examining personal distress, a significant main effect of condition found that participants in the empathy training condition reported significantly less personal distress, or feelings of distress for oneself (*M* = 3.36, *SE* = .28), after exposure to the needy target, compared to participants in the control conditions (*M* = 4.13, *SE* = .28), *F*(1,74) = 4.43, *p* = .04. There was no main effect of Gender or Condition × Gender interaction on personal distress, *ps*>.16. When examining empathic emotions, there were no main effects or interaction, *ps*>.38.

#### Emotional empathy in imagined scenarios

We conducted a separate 2 (Condition) × 2 (Gender) ANCOVA on coded expressions of *emotional* empathy in the imaginary scenarios, controlling for baseline scores. A significant main effect of condition found that the empathy training condition led to more *emotional* empathy (*M* = 4.98, *SE* = .19) compared to the control conditions (*M* = 4.46, *SE* = .16), *F*(1,78) = 4.18, *p* = .04. There were no main effects of Gender nor Condition × Gender interactions on emotional empathy, *ps*>.63. (Note that we report practical empathy results in the section on behaviors.)

### General beliefs

#### Aggressive beliefs

A significant Condition × Gender interaction was found on post-intervention aggressive beliefs, controlling for baseline levels, *F*(1,76) = 8.43, *p* = .005. Post-hoc least significant difference (LSD) contrast analyses found that men in the control condition (*M* = 2.04, *SE* = .05) reported significantly more aggressive beliefs compared to all other groups (Men _Empathy condition_: *M* = 1.81, SE = .07; Women _Control Condition_: *M* = 1.80, SE = .05; Women _Empathy Condition_: *M* = 1.87, *SE* = .05), *p*s < .05. No other groups differed from each other, *p*s>.26. A marginally significant main effect of Gender found that men (*M* = 1.93, *SE* = .04) tended to report more aggressive beliefs compared to women (*M* = 1.83, *SE* = .03), *F*(1,76) = 3.30, *p* = .07. There was no main effect of condition, *F*(1,76) = 2.26, *p* = .14.

### Traits / self-perceptions

#### Moral principle of care

A 2 (Condition) × 2 (Gender) ANCOVA on post-intervention moral principle of care was found, controlling for baseline levels, *F*(1,77) = 4.19, *p* = .04. Post-hoc LSD contrast analyses found that this was because men in the empathy training condition (*M* = 4.06, *SE* = .09) scored lower in principle of care than women in the empathy training condition (*M* = 4.29, *SE* = .06), *p* = .04. There were no other significant differences between the four groups (Male control: *M* = 4.23, SE = .07; Female control: *M* = 4.17, *SE* = .07), *p*>.12. There was no main effect of Gender, *F*(1,77) = 1.39, *p* = .24, or Condition, *F*(1,77) = .17, *p* = .68.

#### Dispositional empathy

We next conducted 2 (Condition) × 2 (Gender) ANCOVAs on each of the four subscales of the Davis Interpersonal Reactivity Index at post-intervention, controlling for baseline levels of these traits. When examining Personal Distress, a significant main effect of Gender, *F*(1,78) = 5.18, *p* = .03, found that women (*M* = 2.61, *SE* = .06) scored higher in personal distress than men (*M* = 2.40, *SE* = .08). There was no main effect of Condition or a Condition × Gender interaction on personal distress, *p*s>.44.

When examining Empathic Concern, a marginal main effect of gender, *F*(1,78) = 3.25, *p* = .08, found that women (*M* = 3.84, *SE* = .05) reported marginally greater empathic concern than men (*M* = 3.69, *SE* = .07). A significant main effect Condition on Empathic Concern was also found, but in the opposite direction than was expected, *F*(1,78) = 8.43, *p* = .005. Participants in the empathy training condition (*M* = 3.64, *SE* = .06), scored *lower* on Empathic Concern than those in the control conditions (*M* = 3.88, *SE* = .05). There was no Condition × Gender interaction on Empathic Concern, *F*(1,78) = .37, *p* = .55.

There were no main effects of Condition or Gender, or Condition × Gender interactions for the Perspective Taking and Fantasy subscales, *p*s>.37.

### Behaviors (self-reported and observed)

#### Ratio of giving to receiving social support

A significant Condition × Gender interaction was found on the post-intervention ratio of giving social support to receiving social support, controlling for baseline levels of this ratio, *F*(1,67) = 4.60, *p* = .04. Post-hoc LSD contrast analyses found that condition influenced the giving/receiving ratio in men, but not in women. Men who were in the empathy training condition had higher giving/receiving ratios (*M* = 1.10, *SE* = .10) than men in the control conditions (*M* = 0.84, *SE* = .07), *p* = .03. Women’s giving/receiving ratios were unaffected by condition (Control: *M* = .94, SE = .06; Empathy: *M* = .88, *SE* = .06), *p* = .56. In addition, men in the empathy condition had marginally higher giving/receiving ratios than women in the empathy condition, *p* = .06. No other simple effects were significant in the interaction, *p*s>.15. There was no main effect of Gender, *F*(1,67) = .68, *p* = .41, or Condition, *F*(1,67) = 2.09, *p* = .15, on support ratios.

#### Helping behavioral intentions

On average, 73% of participants offered to volunteer for the anti-bullying organization after watching the distressed person being bullied during the second lab session, and a logistic regression found that this was unrelated to Condition, Gender, or their interaction, *ps*>.82. However, among those who chose to help, a marginal main effect of condition found that participants in the high empathy condition (*M* = 4.06, *SE* = .26) had slightly higher willingness to donate more hours of volunteering to the organization than participants in the control conditions (*M* = 3.47, *SE* = .23), *F*(1,49) = 2.88, *p* = .096. There was no main effect of gender or Condition × Gender interaction on the number of volunteer hours among participants who offered to help, *ps*>.12.

#### Observer-reported empathy

Research assistants rated participants’ empathy levels after their brief interactions in the lab room. They were unaware of participants’ condition and helping behaviors when they provided this rating. This analysis was similar as the prior analyses except that we also included RA gender as a moderator, because of the possibility that RAs would rate participants of the same sex as more empathic. Thus, a 3-way 2 (Condition) × 2 (Participant Gender) × 2(RA Gender) ANCOVA was conducted. A marginally significant main effect of condition was found: research assistants rated participants in the empathy training condition as marginally more empathic (*M* = 5.62, *SE* = .21) compared to participants in the control conditions (*M* = 5.15, *SE* = .28), *F*(1,71) = 2.75, *p* = .10. All other main effects and interactions were non-significant, *ps*>.16.

We also calculated a change score, which subtracted the RA-rated baseline empathy scores from the post-intervention scores. This score might be more appropriate considering that participants were rated as high in empathy, overall. Thus, changes in how people rate them before versus after the intervention might be more relevant. A 3-way 2 (Condition) × 2 (Participant Gender) × 2(RA Gender) ANOVA was conducted on this change score. A main effect of condition found that participants in the empathy training condition (*M* = 1.22) had a larger increase in observer-rated empathy than participants in the control conditions (*M* = -.01), *F*(1,72) = 9.42, *p* = .003. There was a marginal Condition × RA gender, *F*(1,72) = 2.75, *p* = .10, such that male RAs on average saw more increases in empathy in the empathy condition (M_empathy_ = 1.69 versus M_control_ = -0.20), compared to female RAs (M_empathy_ = 0.75 versus M_control_ = 0.19). No other effects emerged as significant, *ps*>.28.

#### Practical empathy in imagined scenarios

A main effect of condition found that the empathy training condition led to marginally *less* practical empathy (*M* = .33, *SE* = .13) compared to the control conditions (*M* = .63, *SE* = .11), *F*(1,78) = 3.26, *p* = .075. There were no main effects of Gender nor was there a Condition × Gender interaction on practical empathy, *ps*>.22.

### Covert follow-up (Time 3) Measures

We examined whether there was an effect of condition or gender on the tendency to respond to the hostile text message (1 = *yes*, 0 = *no*) sent about 6 months after participants were in the lab. There was no effect of either condition, *p* = .46, or gender, *p* = .74, suggesting that the missing (non-response) values were unrelated to our key predictors. Thus, listwise deletion may be an appropriate method of dealing with the missing data, even though it reduces our sample size substantially (from 79 to 43).

A 2(Condition) × 2(Gender) ANOVA tested participants’ coded responses to the hostile text message (higher = more prosocial). A main effect of Gender found that women sent more prosocial responses than men (*Women* = 2.25, *SE* = .11; *Men* = 1.78, *SE* = .13), *F*(1,39) = 7.59, *p* = .009. In addition, a main effect of Condition found that those in the high empathy condition (*M* = 2.25, *SE* = .12) sent more prosocial (less hostile) responses than those in the control conditions (*M* = 1.78, *SE* = .12), *F*(1,39) = 7.59, *p* = .009. The interaction of Gender × Condition was not significant, *p* = .87. These main effects remained significant even after controlling for the number of months since the study, suggesting that the behavioral effects of the empathy training did not fade over time, and that we were able to change habitual responses. Indeed, the correlation between the coded response and the number of months was *r* = .06 (*p* = .80) in the control conditions and *r* = -.06 (*p* = .81) in the empathy condition.

### Overt follow-up (Time 4) Measures: Affective (motives and emotions)

#### Feelings of social connectedness

The analysis revealed no main effect of Condition on feelings of social connectedness, *F*(1,56) = 2.57, *p* = .11, but Gender did have a significant effect, such that men (*M* = 3.60, *SE* = .21) felt more socially connected on average compared to women (*M* = 3.17, *SE* = .17), *F*(1,56) = 6.19, *p* = .02. However, this was qualified by a significant interaction, *F*(1,56) = 4.09, *p* = .05. When splitting by Gender, there was no effect of Condition on women, *F*(1,33) = .14, *p* = .72, but there was a significant Condition effect on men such that they felt more connected in the high empathy condition (*M* = 4.20, *SE* = .32) compared to the control conditions (*M* = 3.00, *SE* = .26), *F*(1,23) = 8.42, *p* = .008.

#### Motives for volunteering

An ANOVA examined the effect of Condition and Gender on participants’ motives for volunteering. We did not control for baseline motives because the baseline measures used an open-ended format that did not directly correspond with the standardized scale used in the overt follow-up. (However, results remain similar when controlling for baseline motives, coded as 1 if present and 0 if absent). There were no significant main effects nor interactions on either other-oriented, *ps*>.20, or self-oriented motives, *ps*>.52.

### Traits / self-perceptions

#### Dispositional empathy

Separate 2(Condition) × 2(Gender) ANOVAs were conducted on the four dispositional empathy subscales. We did not control for baseline levels because a modified version of the scale was used in which participants reported their empathic tendencies *compared to people their own age*. (However, results remain similar when controlling for baseline trait empathy scores.) A marginal main effect of gender found that women (*M* = 2.68, *SE* = .13) scored higher in Personal Distress than men (*M* = 2.35, *SE* = .16), *F*(1,56) = 2.79, *p* = .10. There was no main effect of Condition nor was there a Condition × Gender interaction on Personal Distress, *ps*>.31. There were no significant main effects or interactions for Perspective Taking, *ps*>.11, Empathic Concern, *ps*>.16, and Fantasy, *ps*>.55.

### Behaviors (self-reported and observed)

#### Number of in-person social interactions

A main effect of Condition on social interactions found that participants in the empathy training condition (*M* = 5.84, *SE* = .67) reported talking to more people face-to-face in the past 2 hours than those in the control conditions (*M* = 3.13, *SE* = .60), *F*(1,56) = 8.77, *p* = .004. There was no main effect of Gender, *F*(1,56) = .03, *p* = .85, or interaction, *F*(1,56) = .97, *p* = .33.

#### Ratio of giving to receiving social support

An 2(Condition) × 2(Gender) ANCOVA conducted on the ratio of giving social support to receiving social support, controlling for the baseline support ratio found no significant main effects or interaction, *ps*>.44.

#### Social dilemma game

We examined the effect of Gender and Condition on the tendency to cooperate (coded as 1) versus defect (0) in the social dilemma game (75% of participants cooperated). No effects emerged as significant, *ps*>.11.

## Discussion

This study is among the first that takes a multidimensional approach toward empathy and its correlates, examining how a daily intervention program delivered by text message, *Text to Connect*, can affect the affective, self-perception, and (self-reported and observed) behavioral aspects and implications of empathy (See S2 Table for a detailed summary of the results). This multidimensional framework proves interesting and important in light of the mixed findings. Overall, the empathy messages seemed to have the strongest effects in the domain of (self-reported and observed) *behaviors*, with the most notable findings being that participants in the empathy condition reported that they would volunteer marginally more hours to help a person in distress, were rated by observers as more empathic, responded more prosocially to a stranger who sent them a hostile text message (covert follow-up), and reported more face-to-face social interactions 6 months after the intervention. These behavioral changes are important in themselves, because many would agree that kinder and more socially connected society is desirable to build. Yet there are many reasons to behave prosocially, including self-serving ones (e.g. to look good, to have others return the favor, to bask in the warm glow of giving), and proponents of a kinder and more socially connected society would likely want the kindness to run deeper.

That is why we also assessed more immediate and affective empathic responses. Arguably, it is more empathic for people to help others because they care about others’ well-being and want to alleviate their suffering, not simply for more strategic reasons. In this study, the empathy messages also produced some change in this more immediate, affective domain. For example, participants in the empathy condition reported more prosocial, and less selfish, motives for their ongoing volunteering activities compared to those in the control conditions. Moreover, although participants did not necessarily report feeling more distress on behalf of a needy target (i.e. empathic concern), they did have lower personal distress feelings. This is notable since prior research has found that high personal distress can inhibit helping behavior [105]. In addition, in the domain of general beliefs, males in the empathy condition were also less likely to believe that aggression was acceptable. Taken together these pilot study results suggest that the empathy-training intervention could contribute to more prosocial emotional responses, motives, and (self-reported and observed) behaviors.

Yet, what is perhaps most interesting about the findings is that participants in the empathy condition had significantly lower empathic concern. This is surprising because it makes sense that the various domains of empathy and broader prosociality would fit together more consistently. Although we did not predict these results in advance, it is possible that these relatively lower self-perceptions of empathy stem from either contrast effects [106, 107] or frame of reference effects [108–110]. Both of these processes could lead to a decrease in self-perceived empathy even when other evidence (e.g. observer effects, behavioral intentions) suggests otherwise. For example, with respect to contrast effects, it is possible that when exposed to six empathy-building text messages per day, participants became more aware of the high standard of empathy and compassion, and how much they personally deviate from this standard. Perhaps they occasionally found the messages annoying or refused to follow through on them, and if so, this might have led to self-perceptions of being less than empathic. Indeed, some research finds that people who are asked to recall many (twelve) examples of their assertive behavior see themselves as *less* assertive than those who are asked to recall fewer (six) examples—even though those who are asked to recall more actually do so [111]. This suggests that it is possible that intensive daily reminders of a trait could ironically lead to lowered self-perceptions of that trait even as it increases trait-relevant behaviors. Future studies should attempt better understand the psychological processes that created these contrast effects in self-judgments.

With respect to frame of reference effects, it is possible that the intensive daily reminders of empathy made participants believe that most people were more empathic than them. This altered reference group could have made them see themselves as less empathic in comparison. We tried to address this interpretation by asking participants to respond to the identical dispositional Empathic Concern items during the overt follow-up phase, except that we specified the reference group to be “other people your age.” We found that there were no longer any effects of condition on empathic traits in the follow-up phase, suggesting that reference group effects might have partially explained the results.

In any case, although the lowered self-perceptions of empathy were not expected, they can make us more confident that our results cannot simply be explained by socially desirable responding. If that were the case, then participants likely would have consistently reported more empathic and prosocial traits in the empathy training condition. Instead, participants specifically reported lower self-perceived dispositional empathic concern. When it comes to beliefs (i.e. that aggression is acceptable), motivations (i.e. for volunteering), support provision, and other self-reported and observed behaviors, the results are more consistent–empathy training increases participants’ prosociality.

Notably, empathy training did not influence whether participants would cooperate with their fellow participants in a social dilemma game, and this might be because cooperation with an abstract partner who is not in need is not necessarily the same as empathizing with someone in distress. This can help to point to the boundary limits of Text to Connect–it may be better at affecting more central aspects of empathy related to others’ distress and needs than more peripheral domains of prosocial behavior.

Empathy research is rife with definitional and measurement problems, which is in part why we included a number of different operationalizations and measures. This paper cannot resolve these issues, but can point to different general domains of empathy (immediate affective / motivational components versus more chronic traits) and different implications for different types of behaviors (i.e. empathy-based forms of prosocial behavior versus more general forms such as cooperation).

In terms of gender, it is notable that there were not consistent main effects of gender, despite the fact that many of these measures are known to be associated with gender. To some extent, this could be explained by the fact that the analyses controlled for baseline scores on the measures. There were also no consistent interactions between gender and condition; yet, the few significant interactions found that the empathy training program seemed to be more effective for males on these measures. It remains unclear why the program worked better for males on some measures but not others. Perhaps this may be pursued with brain imaging approaches which have indicated that females and males rely on divergent processing strategies when solving emotional tasks [112]. Specifically, females have been shown to recruit more emotion and self-related regions, whereas males activate more cortical cognition areas which may be more affected by the strategies in *Text to Connect*.

### Theoretical and applied implications

This paper makes a number of contributions. Theoretically, it contributes to a literature that often relies on correlational or longitudinal data because many scholars see empathy as a relatively stable disposition that is therefore not amenable to change. Correlational research makes it impossible to know the directionality of the effects, such as if empathy causes increased prosocial behaviors, decreased aggression, and better health and well-being, or if such outcomes instead lead to higher empathy. Both types of studies are also subject to additional third variables that may better explain the correlations with empathy (e.g. social desirability, socioeconomic variables). Our experimental approach can address these problems.

Our results suggest that empathy is multidimensional and that an experimental intervention can differentially impact various facets and correlates of empathy. Notably, we find that affective measures of empathy and prosocial behaviors appear to be more responsive to experimental influence than trait measures of personality. In terms of trying to increase prosociality in the real world, shifting empathic behavior and tendencies may have a more direct positive impact than merely shifting self-perceptions of empathic traits. This is consistent with theory and practice in modern behavioral intervention that verbally mediated beliefs may differ from real contingencies that take place at the present moment (e.g., [113]).

Practically, we have created an inexpensive, easy to administer program, *Text to Connect*, that promotes empathic motivations and prosocial behaviors among college students and can be widely distributed and implemented within day-to-day natural activities. After future research determines appropriate dosage levels that limit contrast effects on empathic self-perceptions, there may be many practical applications for such a program, with the caveat that additional studies are needed to examine the efficacy of such messages among other populations. For example, this program could potentially be helpful in reducing aggression or bullying behavior in high-risk adolescents/emerging adults or other groups (e.g. narcissistic people [114]).

In addition, empathy has been shown to be associated with a number of benefits in the workplace [115–117]. Thus, a similar text messaging program could be used to help train managers to take the perspective of their employees—something that is inhibited when in positions of power [118]. This program could also be adapted to help train doctors, therapists, and social workers to be more empathic of their patients’ needs, since research has found that empathy in mental and physical health professionals is associated with better patient outcomes [20, 119–123].

Finally, this program could also be adapted to help increase young adult parents’ sensitivity to their child’s cues, since sensitive parenting predicts healthier future child outcomes [124–127], reduced antisocial behavior and callous unemotional traits among youth [128], and also potentially improved outcomes for offspring of parents with mental health issues [129–131].

### Strengths, limitations, future directions

Our paper describes the development and initial pilot evidence for the efficacy of a text message based empathy-building intervention. The six month double-blind follow up demonstrates that there can be relatively long-term benefits of this intervention; however, future research is needed with longer follow-up periods and larger samples. Also, in future research we will continue to refine the messages and optimal intervention dosage to further explore the potential impact. This is the first known study to try to create changes in empathy using text messages. Although the study finds that more immediate and concrete outcomes (e.g. self-reported and observed behaviors) were more consistent than more abstract dispositional-level ones, this disconnect in itself is interesting and warrants future research. Since most of the measures in the current study relied on self-reports, more behavioral measures are need to properly assess Text to Connect in the future.

Several other limitations remain. For example, because of our limited funding, we were only able to collect data from a relatively small sample. This small sample size led to low statistical power, limiting our ability to test for the difference between both types of control groups with the intervention group. However, we consider this study a pilot test, and will conduct larger randomized control trials in the future. We urge readers to consider the results presented using the Cohen’s D effect sizes, which are reported in S2 Table. Unlike p-values, effect sizes are not dependent upon sample sizes, and thus, it makes sense in the context of a small pilot study to use them as a more reliable guide.

The potential for false positives (Type I errors) must be considered in this study. Since there were 28 dependent measures, and we expect that 1 in 20 (p-value = .05) will be a false positive, it is likely that at least one of our results is a false positive when considering all of these results together. However, the main result that a brief messaging intervention can affect some types of empathic habits appears robust, and one of the purposes of our study was to narrow down the specific types of empathy and related outcomes that would be most affected by *Text to Connect*.

Still, in S2 Table, we used the Benjamini-Hochberg test to calculate the false positive probability of each outcome [132]. When setting the false positive rate at 15%, which is reasonable for an exploratory study, all results that were statistically significant at p < .05 had a false positive rate that was below 15%. Future studies should include larger sample sizes, but we report the results of *Text to Connect* as is in the interests of avoiding false negatives (Type II errors) in a pilot study testing a novel, potentially beneficial intervention.

Another potential limitation is that the sample mainly consisted of college students. Perhaps this group should be targeted because research has found declines in empathy among them [7] and because empathy is relatively lower among higher socioeconomic status groups compared to lower socioeconomic status groups [133, 134]. However, it is unclear if these results would generalize to other populations, thus posing an additional avenue for future research.

In addition, we did not have any way to measure whether participants opened the text messages or read them. We also did not ask participants if they actually implemented the instructions. There were several reasons for this. First, participant burden in this study was already high. They received 6 intervention messages daily and also received 6 data collection (e.g. mood) questions each day. They then responded to those data collection measures. This is a total of 18 messages daily between the researchers and the participants. Adding a question about whether they implemented the instruction, plus their response, would increase this to a total of 30 messages per day, which is an undue burden. In addition, we did not ask participants whether they followed the instructions because we were trying to design a program that could be implemented and disseminated in real world settings if there was evidence that it worked. Asking people whether they followed the instructions is not naturalistic and also shifts the motivation more extrinsically and less intrinsically. Thus, it remains unclear if the effects exist because participants simply know they are receiving empathic instructions, because they read the full instructions, and / or because they act on them. However, we do know that participants read the mood / social interaction question that came 5 minutes after the intervention text message over 90% of the time, making it plausible that participants also read the message that came before it.

It also remains unclear whether the behavioral, cognitive, or emotional aspects of the intervention contributed to the results, and future studies should disentangle these three components of *Text to Connect* to determine the extent to which they each independently contribute to increase empathy and prosocial behavior. We suspect that messages that address emotional aspects of empathy would be more effective than cognitive or behavioral ones alone. Emotional aspects of empathy tap into altruistic motivation (i.e. the desire to help others), while cognitive (e.g. taking others’ perspectives) and behavioral (e.g. helping others) aspects of empathy can both be motivated by more self-oriented goals.

Future research should also examine whether the dosage matters. Participants in the current study received 6 messages a day, which is admittedly a fairly intensive approach. We chose this relatively high frequency of messages because we were attempting to create empathic habits-of-mind. However, it may have had the unintended side effect of making participants see themselves as less empathic when they noticed themselves deviating from such a high standard. It is possible that a lower dosage would have the same positive consequences as the current study, without creating the ironic changes in empathic self-perceptions. This is a similar rationale as in pharmaceutical trials, where it is desirable to determine the smallest dosage possible in order to achieve the desired results without side effects. Yet to date, nearly all published mobile intervention trials do not vary dosage, but simply compare receiving one type of intervention to a control group [72], as in the current study.

Another potential future direction of this research would be into the physiological and brain mechanisms that may underlie the empathy intervention. A growing literature has addressed the brain mechanisms of empathy [135, 136]. Recent work suggests that brain areas that respond to baby stimuli overlap with some of these empathy regulation areas [137, 138]. For example, one study showed that dimensions of empathy were related to certain brain responses in a parent-response task [139]. Indeed, empathy figures prominently in the NIMH research domain for social processes and the construct of understanding others, with the belief that better understanding the brain mechanisms is central to addressing mental health concerns [140, 141] including depression [142]. With respect to parenting, we speculate that understanding and optimizing empathy among parents may lead to higher empathy in subsequent generations, with the potential for related social benefits.

Finally, mobile intervention studies rarely compare the strength of mobile interventions to actual face-to-face interventions of a comparable nature [72]; yet, it is important to do so in order to determine which type of intervention is most effective. As reviewed in the introduction, there are a number of effective ways to increase empathy using face-to-face methods. Considering the interpersonal nature of empathy, it may be even more desirable to use such methods whenever possible. Moreover, it is very likely that those more emotionally evocative, face-to-face, interventions would be more effective at making people more empathic. However, it is not always possible to conduct resource-intensive face-to-face interventions, and mobile interventions can be effective in these cases.

### Conclusion

Strong social connections are critical to humans’ health and well-being [1, 2], and it is important to find ways to help people develop their ability to feel compassion and give care to others. Although at times mobile technologies may seem to disconnect us from each other [9, 10], in this study we find that they can also be used to increase (self-reported and observed) prosocial behaviors and empathic motivations. We used a theoretically driven approach to build young adults’ prosociality and found that participants who received daily reminders to care about others over a 2 week period had more altruistic motivations and behaviors, even up to 6 months later. Yet surprisingly, they simultaneously rated themselves as less empathic on self-reported traits. Future research will help us to better understand these results. Until then, researchers and practitioners should use this empathy-building intervention with these mixed results in mind, being aware of the potential limits of technological interventions to improve such a fundamentally social tendency.

## Supporting Information

S1 Table Full text of text messages in study. (DOCX)

S2 Table Detailed summary of results. (DOCX)
