# Supplementary material for: Can Text Messages Increase Empathy and Prosocial Behavior? The Development and Initial Validation of Text to Connect
Source: PLoS One. 2015 Sep 10;10(9):e0137585. doi: 10.1371/journal.pone.0137585 (PMC4565638; doi:10.1371/journal.pone.0137585)
Supplement: S1 Table — (DOCX) [file pone.0137585.s001.docx]

| **Empathy Condition** | **Self/Objective (Control) Condition** |  |
| --- | --- | --- |
| Send a nice text message to someone close right now. Try to make them feel loved. | Reply to this text message describing the nearest object to you. | **Behavioral** |
| During your social interactions today, try to avoid distractions, give eye contact, and face the person you are talking to. | During your social interactions today, keep track of time, and don't get too caught up in frivolous interactions. |  |
| Think about somebody close to you. Do a small nice thing for this person today. | Think about all that you deserve. Do something nice for yourself today. |  |
| Smile at the next person you see, no matter who they are. | Smile at the mirror or window next time you see your reflection. |  |
| Use active listening phrases during your social interactions today: e.g. "What I hear you saying is…" | During your social interactions today, make sure that you share your own views in this conversation. Don’t let other people dominate. |  |
| Imagine what the last person you interacted with was thinking about. What does the world look like from their perspective? | During your last social interaction, think of how you could have been more cool, objective, and logical in the conversation. | **Cognitive** |
| Reflect on somebody close to you. What do they hope to achieve in life? Think of one positive step they have recently taken to achieve their goals. | Recall you last social interaction as if you were a fly on the wall, from an observer perspective. Focus on the facts only. |  |
| Think about your last social interaction. What obstacles or challenges does the person face? See these problems from their point of view. | Think about your last social interaction. Think of two specific ways you could be more neutral and objective in the future. |  |
| Think about your last social interaction. What was important to them in the conversation? Can you try to see the world the way they do? | Think about your last social interaction. Try to analyze what happened in the conversation, and have some healthy distance from it |  |
| Reflect on somebody close to you. Imagine that you are inside their mind. What do you think they are thinking about right now? | Take a few moments to pay attention to what you are currently thinking. What is the next thing you have to do today? |  |
| Think about someone you have recently had trouble getting along with. For the next 30 seconds, focus on what you have in common with this person. | Think about someone you have recently had trouble getting along with. For the next 30 seconds, think of a good counter argument to prove your point. |  |
| Think about someone you have recently had trouble getting along with. For the next 30 seconds, try to understand their point of view. | Think about someone you have recently had trouble getting along with. For the next 30 seconds, think of how you might make them see your point of view. |  |
| Think about your last social interaction. What was your interaction partner really trying to say? Did you accurately read their body language? | Think about your last social interaction. What were you really trying to say. Did the other person accurately read your body language? |  |
| Reflect on somebody close to you. What is the most recent thing in their life that made them feel sad or disappointed? How can you be there for them? | Reflect on this question. When was the most recent time when people loved to hear your stories, jokes, or ideas? How good did it make you feel? | **Emotional** |
| Think about someone you know who might be feeling down. Try to feel their pain. Imagine what else they are feeling. | Think about a time that you felt powerful, like you had an influence over others. Try to remember how good this felt. |  |
| Think about the last time a loved one was upset. Try to understand his or her feelings. | Think about the last time you felt proud. Try to remember all your positive feelings. |  |
| Reflect on somebody close to you. Send this person warm thoughts of unconditional love and acceptance. | For the next 30 seconds, focus on who you are as a unique individual and what makes you special. |  |
| Think about someone close to you. How can you show this person that you accept them and care for them? | Think about your last social interaction. Did this person give you the level of respect you deserve? |  |
| Think about someone you know who has just received some good news. Try to feel their joy. Do they have any other feelings? | Think about the last time you experienced a success. Try to recall your good feelings. How can you have more successes like this? |  |
| Reflect on somebody close to you. What makes them feel happiest? | Reflect on this question. What makes you better than others? What are your unique skills and qualities? |  |
| Focus on connecting with and feeling close to the person you last interacted with, no matter who they are. | Focus on the ways that you are different and unique from the person you last interacted with, no matter who they are. |  |

© 2013
