# Supplementary material for: Can Text Messages Increase Empathy and Prosocial Behavior? The Development and Initial Validation of Text to Connect
Source: PLoS One. 2015 Sep 10;10(9):e0137585. doi: 10.1371/journal.pone.0137585 (PMC4565638; doi:10.1371/journal.pone.0137585)
Supplement: S2 Table — (DOCX) [file pone.0137585.s002.docx]

| Measure | Empathy condition | | Combined Control conditions | | Statistical tests | Effect Size (Cohen’s d) | Benjamini-Hochberg significance | Benjamini-Hochberg false discovery probability^ |
| --- | --- | --- | --- | --- | --- | --- | --- | --- |
|  | *Men* | *Women* | *Men* | *Women* |  |  |  |  |
| **Immediate Post-Intervention (Time 2)** |  |  |  |  |  |  |  |  |
| *Affective (motives and emotions)* |  |  |  |  |  |  |  |  |
| Motives for volunteering: To help others | 92.2% (12.4%) | 83.5% (7.7%) | 74.0% (8.0%) | 63.8% (7.4%) | Condition: *F*(1,65)=4.39, *p*=.04 | Condition: .51 | Significant | 0.112 |
|  |  |  |  |  | Gender: *F*(1,65)=1.10, *p*=.30 | Gender: .26 |  |  |
|  |  |  |  |  | Interaction: *F*(1,65)=.01, *p*=.94 |  |  |  |
| Motives for volunteering: To feel good | 28.3% (13.4%) | 27.3% (8.3%) | 44.1% (8.7%) | 52.8% (8.1%) | Condition: *F*(1,65)=4.41, *p*=.04 | Condition: d=.51 | Significant | 0.112 |
|  |  |  |  |  | Gender: *F*(1,65)=.15, *p*=.70 | Gender: d=.10 |  |  |
|  |  |  |  |  | Interaction: *F*(1,65)=.24, *p*=.63 |  |  |  |
| Motives for volunteering:To benefit career | 0.5% (12.2%) | 29.2% (7.6%) | 13.7% (8.0%) | 32.8% (7.4%) | Condition: *F*(1,65)=.87, *p*=.35 | Condition: d=.23 | Not significant | 0.6125 |
|  |  |  |  |  | Gender: *F*(1,65)=7.00, *p*=.01 | Gender: d=.65 |  |  |
|  |  |  |  |  | Interaction: *F*(1,65)=.28, *p*=.60 |  |  |  |
| Personal distress emotions in response to target in distress | 2.96 (.45) | 3.76 (.32) | 4.02 (.36) | 4.23 (.32) | Condition: *F*(1,74)=4.43, *p*=.04 | Condition: d=.48 | Significant | 0.112 |
|  |  |  |  |  | Gender: *F*(1,74)=1.92, *p*=.17 | Gender: d=.32 |  |  |
|  |  |  |  |  | Interaction: *F*(1,74)=.66, *p*=.42 |  |  |  |
| Empathic emotions in response to target in distress | 5.86 (.29) | 5.79 (.20) | 5.94 (.23) | 6.12 (.20) | Condition: *F*(1,74)=.76, *p*=.39 | Condition: d=.20 | Not significant | 0.642 |
|  |  |  |  |  | Gender: *F*(1,74)=.05, *p*=.82 | Gender: d=.05 |  |  |
|  |  |  |  |  | Interaction: *F*(1,74)=.28, *p*=.60 |  |  |  |
| Emotional empathy in imagined scenarios (e.g. emotional resonance, acknowledging emotion) | 4.99 (.32) | 4.97 (.22) | 4.35 (.24) | 4.58 (.22) | Condition: *F*(1,78)=4.18, *p*=.04 | Condition: d=.45 | Significant | 0.112 |
|  |  |  |  |  | Gender: *F*(1,78)=.16, *p*=.69 | Gender: d=.09 |  |  |
|  |  |  |  |  | Interaction: *F*(1,78)=.23, *p*=.63 |  |  |  |
| *General beliefs* |  |  |  |  |  |  |  |  |
| Aggressive beliefs | 1.81 (.07) | 1.87 (.05) | 2.04 (.05) | 1.80 (.05) | Condition: *F*(1,76)=2.26, *p*=.14 | Condition: d=.34 | Significant (males) | 0.065 |
|  |  |  |  |  | Gender: *F*(1,76)=3.30, *p*=.07 | Gender: d=.41 |  |  |
|  |  |  |  |  | Interaction: *F*(1,76)=8.43, *p*=.005 | Men: d=1.06 |  |  |
|  |  |  |  |  |  | Women: d=.31 |  |  |
| *Traits / self-perceptions* |  |  |  |  |  |  |  |  |
| Moral principle of care | 4.06 (.09) | 4.29 (.06) | 4.23 (.07) | 4.17 (.07) | Condition: *F*(1,77)=.17, *p*=.68 | Condition: d=.09 | Not significant | 0.112 |
|  |  |  |  |  | Gender: *F*(1,77)=1.39, *p*=.24 | Gender: d=.27 |  |  |
|  |  |  |  |  | Interaction: *F*(1,77)=4.19, *p*=.04 | Men: d=.51 |  |  |
|  |  |  |  |  |  | Women: d=.38 |  |  |
| Dispositional empathy: Empathic Concern | 3.54 (.11) | 3.74 (.07) | 3.83 (.08) | 3.93 (.07) | Condition: *F*(1,78)=8.43, *p*=.005 | Condition: d=.64 | Significant | 0.065 |
|  |  |  |  |  | Gender: *F*(1,78)=3.25, *p*=.08 | Gender: d=.40 |  |  |
|  |  |  |  |  | Interaction: *F*(1,78)=.37, *p*=.55 |  |  |  |
| Dispositional empathy: Perspective Taking | 3.68 (.13) | 3.58 (.09) | 3.63 (.10) | 3.72 (.09) | Condition: *F*(1,78)=.24, *p*=.63 | Condition: d=.11 | Not significant | 0.840 |
|  |  |  |  |  | Gender: *F*(1,78)=.002, *p*=.97 | Gender: d=.01 |  |  |
|  |  |  |  |  | Interaction: *F*(1,78)=.79, *p*=.38 |  |  |  |
| Dispositional empathy: Fantasy | 3.28 (.14) | 3.32 (.10) | 3.39 (.10) | 3.38 (.10) | Condition: *F*(1,78)=.60, *p*=.44 | Condition: d=.17 | Not significant | 0.684 |
|  |  |  |  |  | Gender: *F*(1,78)=.02, *p*=.90 | Gender: d=.03 |  |  |
|  |  |  |  |  | Interaction: *F*(1,78)=.05, *p*=.82 |  |  |  |
| Dispositional empathy: Personal Distress | 2.39 (.12) | 2.68 (.08) | 2.41 (.09) | 2.55 (.08) | Condition: *F*(1,78)=.29, *p*=.59 | Condition: d=.12 | Not significant | 0.840 |
|  |  |  |  |  | Gender: *F*(1,78)=5.18, *p*=.03 | Gender: d=.51 |  |  |
|  |  |  |  |  | Interaction: *F*(1,78)=.57, *p*=.45 |  |  |  |
| *Behaviors (self-reported and observed)* |  |  |  |  |  |  |  |  |
| Ratio of giving to receiving social support | 1.11 (.10) | .88 (.06) | .84 (.07) | .94 (.06) | Condition: *F*(1,67)=2.09, *p*=.15 | Condition: d=.34 | Significant (males) | 0.112 |
|  |  |  |  |  | Gender: *F*(1,67)=.68, *p*=.41 | Gender: d=.20 |  |  |
|  |  |  |  |  | Interaction: *F*(1,67)=4.60, *p*=.04 | Men: d=.91 |  |  |
|  |  |  |  |  |  | Women: d=.18 |  |  |
| Helping behavior (1=yes, 0=no) in response to target in distress | 71.3% (11.4%) | 72.3% (7.9%) | 75.2% (9.0%) | 72.3% (7.9%) | Condition: *F*(1,76)=.01, *p*=.92 | Condition: d=.02 | Not significant | 0.954 |
|  |  |  |  |  | Gender: *F*(1,76)=.05, *p*=.83 | Gender: d=.05 |  |  |
|  |  |  |  |  | Interaction: *F*(1,76)=.05, *p*=.83 |  |  |  |
| Hours of offered help in response to target in distress | 4.37 (.41) | 3.75 (.32) | 3.23 (.36) | 3.71 (.29) | Condition: *F*(1,49)=2.88, *p*=.10 | Condition: d=.46 | Not significant | 0.205 |
|  |  |  |  |  | Gender: *F*(1,49)=.05, *p*=.83 | Gender: d=.06 |  |  |
|  |  |  |  |  | Interaction: *F*(1,49)=2.50, *p*=.12 |  |  |  |
| Observer-reported empathy | 5.64 (.35) | 5.60 (.24) | 4.90 (.28) | 5.39 (.25) | Condition: *F*(1,71)=2.75, *p*=.10 | Condition: d=.37 | Not significant | 0.205 |
|  |  |  |  |  | Gender: *F*(1,71)=.65, *p*=.42 | Gender: d=.19 |  |  |
|  |  |  |  |  | Interaction: *F*(1,71)=.96, *p*=.33 |  |  |  |
| Practical empathy in imagined scenarios (e.g. offering to listen or help) | .33 (.21) | .33 (.15) | .43 (.16) | .84 (.15) | Condition: *F*(1,78)=3.26, *p*=.075 | Condition: d=.40 | Not significant | 0.191 |
|  |  |  |  |  | Gender: *F*(1,78)=1.43, *p*=.24 | Gender: d=.27 |  |  |
|  |  |  |  |  | Interaction: *F*(1,78)=1.47, *p*=.23 |  |  |  |
| **Covert follow-up (Time 3)** |  |  |  |  |  |  |  |  |
| Responses to hostile text message from stranger (higher = more prosocial) | 1.50 (.23) | 1.91 (.16) | 1.05 (.18) | 1.46 (.16) | Condition: *F*(1,75)=6.14, *p*=.02 | Condition: d=.56 | Significant | 0.112 |
|  |  |  |  |  | Gender: *F*(1,75)=5.06, *p*=.03 | Gender: d=.52 |  |  |
|  |  |  |  |  | Interaction: *F*(1,75)=.00, *p*=.99 |  |  |  |
| **Overt follow-up (Time 4)** |  |  |  |  |  |  |  |  |
| *Affective (motives and emotions)* |  |  |  |  |  |  |  |  |
| Feelings of social connectedness | 4.20 (.32) | 3.24 (.24) | 3.00 (.26) | 3.11 (.24) | Condition: *F*(1,56)=2.57, *p*=.11 | Condition: d=.42 | Not significant | 0.205 |
|  |  |  |  |  | Gender: *F*(1,56)=6.19, *p*=.02 | Gender: d=.65 |  |  |
|  |  |  |  |  | Interaction: *F*(1,56)=4.09, *p*=.05 |  |  |  |
| Motives for volunteering: Other-oriented (using VFI) | 5.30 (.33) | 4.85 (.25) | 4.95 (.28) | 5.21 (.24) | Condition: *F*(1,55)=.00, *p*=.99 | Condition: d=.00 | Not significant | 0.990 |
|  |  |  |  |  | Gender: *F*(1,55)=.11, *p*=.74 | Gender: d=.09 |  |  |
|  |  |  |  |  | Interaction: *F*(1,55)=1.64, *p*=.21 |  |  |  |
| Motives for volunteering: Self-oriented (using VFI) | 4.86 (.31) | 4.61 (.24) | 4.72 (.27) | 4.81 (.23) | Condition: *F*(1,55)=.01, *p*=.92 | Condition: d=.03 | Not significant | 0.954 |
|  |  |  |  |  | Gender: *F*(1,55)=.10, *p*=.75 | Gender: d=.08 |  |  |
|  |  |  |  |  | Interaction: *F*(1,55)=.40, *p*=.53 |  |  |  |
| *Trait s / self-perceptions* |  |  |  |  |  |  |  |  |
| Dispositional empathy: Perspective Taking† | 3.80 (.21) | 3.33 (.16) | 3.67 (.17) | 3.57 (.16) | Condition: *F*(1,56)=.10, *p*=.76 | Condition: d=.08 | Not significant | 0.937 |
|  |  |  |  |  | Gender: *F*(1,56)=2.55, *p*=.12 | Gender: d=.41 |  |  |
|  |  |  |  |  | Interaction: *F*(1,56)=1.13, *p*=.29 |  |  |  |
| Dispositional empathy: Empathic Concern† | 3.76 (.22) | 3.65 (.17) | 3.59 (.18) | 4.00 (.16) | Condition: *F*(1,56)=.24, *p*=.63 | Condition: d=.13 | Not significant | 0.840 |
|  |  |  |  |  | Gender: *F*(1,56)=.68, *p*=.41 | Gender: d=.21 |  |  |
|  |  |  |  |  | Interaction: *F*(1,56)=2.02, *p*=.16 |  |  |  |
| Dispositional empathy: Fantasy† | 3.33 (.27) | 3.52 (.21) | 3.36 (.22) | 3.44 (.20) | Condition: *F*(1,56)=.01, *p*=.91 | Condition: d=.03 | Not significant | 0.954 |
|  |  |  |  |  | Gender: *F*(1,56)=.36, *p*=.55 | Gender: d=.16 |  |  |
|  |  |  |  |  | Interaction: *F*(1,56)=.07, *p*=.79 |  |  |  |
| Dispositional empathy: Personal Distress† | 2.21 (.24) | 2.76 (.19) | 2.48 (.20) | 2.61 (.18) | Condition: *F*(1,56)=.09, *p*=.77 | Condition: d=.08 | Not significant | 0.937 |
|  |  |  |  |  | Gender: *F*(1,56)=2.79, *p*=.10 | Gender: d=.43 |  |  |
|  |  |  |  |  | Interaction: *F*(1,56)=1.04, *p*=.31 |  |  |  |
| *Behaviors (self-reported and observed)* |  |  |  |  |  |  |  |  |
| Number of in-person social interactions | 6.20 (1.09) | 5.47 (.84) | 2.60 (.89) | 3.67 (.81) | Condition: *F*(1,56)=8.77, *p*=.004 | Condition: d=.04 | Significant | 0.0653 |
|  |  |  |  |  | Gender: *F*(1,56)=.03, *p*=.85 | Gender: d=.77 |  |  |
|  |  |  |  |  | Interaction: *F*(1,56)=.97, *p*=.33 |  |  |  |
| Ratio of giving to receiving social support | 1.21 (.19) | .99 (.14) | 1.13 (.15) | 1.10 (.13) | Condition: *F*(1,47)=.01, *p*=.92 | Condition: d=.03 | Significant | 0.112 |
|  |  |  |  |  | Gender: *F*(1,47)=.60, *p*=.44 | Gender: d=.22 |  |  |
|  |  |  |  |  | Interaction: *F*(1,47)=.37, *p*=.55 |  |  |  |
| Social dilemma game with other participant (% cooperating) | 50.0% (13.7%) | 76.5% (10.5%) | 80.0% (11.25) | 83.3% (10.2%) | Condition: *F*(1,56)=2.59, *p*=.11 | Condition: d=.42 | Not significant | 0.205 |
|  |  |  |  |  | Gender: *F*(1,56)=1.69, *p*=.20 | Gender: d=.34 |  |  |
|  |  |  |  |  | Interaction: *F*(1,56)=1.02, *p*=.32 |  |  |  |

|  |
| --- |

Note: Standard errors are in parentheses since baseline scores were added as covariates whenever possible.

† In the overt follow-up, a modified version of the Davis Interpersonal Reactivity Index was administered. Participants were asked to report the extent to which each item applied to them “compared to most people your age.”

^Benjamini-Hochberg false discovery rate was set at 0.15. A false discovery rate of 0.15 implies that up to 15% of the significant results may be due to chance. Results that are below this false discovery rate are deemed as “significant” for the Benjamini-Hochberg test. The exact false discovery rates are located in this column.
